# Authentication of Acori Tatarinowii Rhizoma (*Shi Chang Pu*) and its adulterants by morphological distinction, chemical composition and ITS sequencing

**DOI:** 10.1186/s13020-016-0113-x

**Published:** 2016-09-26

**Authors:** Kelly Yin-Ching Lam, Chuen-Fai Ku, Huai-You Wang, Gallant Kar-Lun Chan, Ping Yao, Huang-Quan Lin, Tina Ting-Xia Dong, Hong-Jie Zhang, Karl Wah-Keung Tsim

**Affiliations:** 1 Division of Life Science, and Center for Chinese Medicine, The Hong Kong University of Science and Technology, Clear Water Bay Road, Hong Kong, China; 2 School of Chinese Medicine, Hong Kong Baptist University, Hong Kong, China; 3 HKUST Shenzhen Research Institute, Hi-Tech Park, Nanshan, Shenzhen, Guangdong Province China

## Abstract

**Background:**

Acori Tatarinowii Rhizoma (ATR; rhizome of *Acorus tatarinowii* Schott) (*Shi Chang Pu*) is widely used in Chinese medicine (CM) to resuscitate, calm the mind, resolve *shi* (*dampness*) and harmonize the *wei* (*stomach*). Seven different species have been identified as belonging to the genus *Acorus*, all of which can be found in China. However, it can be difficult to distinguish the different species of *Acorus* because of their morphological similarities. The aim of this study was to authenticate *Acorus* species using macroscopic and microscopic techniques, chemical analysis and DNA authentication and to compare the resolution power and reliability of these different methods.

**Methods:**

Four batches of ATR, Acori Graminei Rhizoma (AGR), Acori Calami Rhizoma (ACR) and Anemones Altaicae Rhizoma (AAR) (totaling 16 samples) were collected from Hong Kong and mainland China. The major characteristic features of these *Acorus* species were identified by macroscopic and microscopic examination. The identified samples were also analyzed by UHPLC analysis. Principal component analysis (PCA) and hierarchal clustering analysis (HCA) on UHPLC results were used to differentiate between the samples. An internal transcribed spacer (ITS) was selected as a molecular probe and a modified DNA extraction method was developed to obtain trace amounts of DNA from the different *Acorus* species. All extracted DNA sequences were edited by Bioedit and aligned with the ClustalW. And the sequence distances were calculated using the Maximum Parsimony method.

**Results:**

Macroscopic and microscopic analyses allowed for AAR to be readily distinguished from ATR, AGR and ACR. However, it was difficult to distinguish between ATR, AGR and ACR because of their similar morphological features. Chemical profiling revealed that α- and β-asarone were only found in the ATR, AGR and ACR samples, but not in the AAR samples. Furthermore, PCA and HCA allowed for the differentiation of these three species based on their asarone contents. Morphological authentication and chemical profiling allowed for the partial differentiation of ATR, AGR ACR and AAR. DNA analysis was the only method capable of accurately differentiating between all four species.

**Conclusion:**

DNA authentication exhibited higher resolution power and reliability than conventional morphological identification and UHPLC in differentiating between different *Acorus* species.

**Electronic supplementary material:**

The online version of this article (doi:10.1186/s13020-016-0113-x) contains supplementary material, which is available to authorized users.

## Background

The dried rhizome from *Acorus tatarinowii* Schott (*Shi Chang Pu*) is used as a Chinese medicine (CM), which is known as Acori Tatarinowii Rhizoma (ATR). *A. tatarinowii* is recorded in the Chinese pharmacopoeia (2015) as the official botanical source of ATR. ATR is produced in the Sichuan, Zhejiang, Jiangsu and Hunan provinces of China [1]. However, there are seven different species of plant belonging to the *Acorus* genus, which are distributed from the northern temperate areas of the globe to the sub-tropical regions. Notably, all seven of these species can be readily found in China. Three species of *Acorus*, including ATR, Acori Graminei Rhizoma (AGR; rhizome derived from the *A. gramineus* Soland; *Jin Qian Chang Pu*) and Acori Calami Rhizoma (ACR; rhizome derived from the *A. calamus* Linn; *Shui Chang Pu*) are widely used in CM.

The aerial parts and rhizomes of these three plants show similar morphological characteristics, and these plants have been described as herbal medicines in a variety of different national pharmacopoeia in Asia. For example, ATR is used for epilepsy, loss of consciousness, forgetfulness and insomnia in CM [2]. In Korea and Japan [3], AGR is used to treat convulsions and stomach aches, and this material is also used as a sedative in oriental medicine. In contrast, ACR is used to treat cognitive disorders, epilepsy, asthma, pain and diabetes in India [4, 5]. In addition, Anemones Altaicae Rhizoma (AAR; *Jiu Jie Chang Pu*), which is the rhizome from *Anemone altaica* Fisch. ex Mey, is a member of the ranunculaceae family, which has been used as a substitute for ATR to treat dreaminess, amnesia, rheumatoid arthritis and epilepsy [6]. These four CM herbs are all known by the same Chinese name “*Chang Pu*” and share several morphological similarities, making it difficult to differentiate between these plants based on their physical appearance.

The aim of this study was to develop a new method to authenticate *Acorus* species using a combination of macroscopic and microscopic examination, chemical analysis and DNA authentication, as well as comparing the resolution power and reliability of these different methods.

## Methods

### Chemicals, reagents and herbal materials

The chemical standards α- and β-asarone (purity ≥98 %) were purchased from TLCM (Hong Kong, China). Acetonitrile and ethanol (HPLC grade) for UHPLC analysis were purchased from Merck (Darmstadt, Germany). Ultra-pure water was obtained from a Milli-Q purification system (Millipore, Molsheim, France). Four batches of ATR, AGR, ACR and AAR (totaling 16 samples, which were numbered from 1 to 16) were collected from herbal markets in Hong Kong and mainland China (Table 1). All of the samples were authenticated by Dr. Tina Dong [Hong Kong University of Science and Technology (HKUST)], according to their morphological characteristics and the descriptions of these materials found in the Chinese Pharmacopoeia (2015) and related documents [7, 8]. Voucher specimens were collected and deposited at The Centre for Chinese Medicine Research and Development, HKUST, under the accession numbers listed in Table 1.

**Table 1:** ATR, AGR, ACR and AAR collected from different regions of China

Herbs	Sample	Voucher no.	Regions
ATR	1	ATR-1-2014	Liuzhou, Guangxi
	2	ATR-2-2014	Lingbao, Henan
	3	ATR-3-2014	Taiyuan, Shanxi
	4	ATR-4-2014	Tianshui, Gansu
AGR	5	AGR-1-2014	Bozhou, Anhui
	6	AGR-2-2014	Muyang, Jiangsu
	7	AGR-3-2014	Xian, Shanxi
	8	AGR-4-2014	Datian, Fujian
ACR	9	ACR-1-2014	Anxin, Hebei
	10	ACR-2-2014	Suqian, Jiangsu
	11	ACR-3-2014	Xiaoshan, Hangzhou
	12	ACR-4-2014	Muyang, Jiangsu
AAR	13	AAR-1-2014	Jiujiang, Jiangxi
	14	AAR-2-2014	Hanzhong, Shanxi
	15	AAR-3-2014	Zigong, Sichuan
	16	AAR-4-2014	Bozhou, Anhui

### Macroscopic and microscopic examination

The appearance, color, odor and taste characteristics of the samples were observed and recorded, together with digital color photographs of each sample [9]. Representative samples of the transverse section of each batch were fixed in formalin-acetic acid-alcohol for a minimum of 24 h. After fixing, the samples were dehydrated using a series of graded ethanol (50, 70, 80, 90 and 100 %) (Unichem Ltd, Chessington, UK) and xylene (50, 100 %) (VWR International, Lutterworth, UK) solutions, before being embedded in paraffin wax (Leica, Shanghai, China) using a previously reported technique [10]. The embedded materials were then cut into 15-μm-thick sections using a rotary microtome (Leica), and stained with safranin-T (Sigma-Aldrich, St Louis, MO, USA) and fast green FCF solution (Sigma-Aldrich). Finally, the stained sections were sealed with DPX-Mountant (Sigma-Aldrich). At least 20 different transverse sections from each sample were prepared. For the powder sections, samples of each of these crude drugs were powdered using a grinder and passed through a 300-μm sieve. The powdered materials were sealed with dilute glycerine and observed under a ZEISS Axio Scope A1 universal microscope (Zeiss Group, Jena, Germany). The powder of each sample was observed for at least 10 slides. The distinct characteristics of these materials were observed under light and polarized light microscopes using a ZEISS Axio Scope A1 universal microscope equipped with a reflector Axio-photo module (Zeiss Group, Jena, Germany) and a direct current (DC) camera.

### Chemical analysis by UHPLC-DAD

Standard stock solutions were prepared at a concentration of 50 mg/L in ethanol, which was identified as the optimal solvent for the extraction of asarone. One gram of powdered sample material was accurately weighed and placed in a centrifuge tube with 10 mL of ethanol. After sonication in a Branson 5200 sonicator (Branson Ultrasonic Corp, Connecticut, USA) for 30 min, the mixture was centrifuged (Eppendorf centrifuge 5810R, Eppendorf, Hamburg, Germany) at 3000×*g* for 5 min. The supernatant was then transferred to a 25-mL volumetric flask and the extraction process was repeated once more. The combined supernatants were diluted to a total volume of 25 mL with ethanol and filtered through a 0.45-μm PTFE filter before being analyzed. UHPLC-DAD analysis was conducted on a Thermo Scientific™ UltiMate 3000 UHPLC System comprising a vacuum degasser, binary pump, auto-sampler, thermostatted column compartment and DAD (Thermo Fisher Scientific, Waltham, MA, USA), which was used for acquiring chromatograms and UV spectra. The samples were analyzed using a Kinetex C18 column (phenomenex, Torrance, CA, USA; 2.1 × 100 mm, 2.6 μm), which was eluted with a mobile phase consisting of water (A) and acetonitrile (B) according to the following gradient program: 30–45 % (B) 0–5 min; 45 % (B) 5–15 min; and 45–100 % (B) 15–25 min. The flow rate, detection wavelength and column temperature were set at 0.2 mL/min, 270 nm and 40 °C, respectively, with an injection volume of 1.0 μL.

### DNA extraction and ITS sequence analysis

Samples of each plant were ground into a fine powder with a grinder and extracted using a DNeasy^®^Plant Mini Kit (Qiagen, Hilden, Germany) according to the manufacturer’s protocol. DNA quantification was performed using a NanoDrop 2000 Spectrophotometer (Thermo Fisher Scientific). An absorbance (A260/A280) ratio of 1.8 indicated insignificant levels of contamination from unwanted proteins and polysaccharides. Five microliters of genomic DNA (~100 ng) was added to a master mix containing 10× PCR buffer (with Mg^2+^ at a concentration of 1.5 mM containing a bromophenol blue loading dye), 0.5 mM dNTP, 400 nM of the forward and reverse primers (ITS-S, AGG AGA AGT CGT AAC AAG; ITS-AS, GTT TCT TTT CCT CCG CT) [11] and 1.5 U of Taq Polymerase (KAPA Taq DNA Polymerase with dye, KAPA Biosystems, Woburn, MA, USA) for a 50 L PCR reaction. A GeneAmp 9700 thermal cycler (Applied Biosystems, Foster City, CA, USA) was used with the following program: 3 min at 95 °C, 30 s at 55 °C and 45 s at 72 °C, followed by 40 cycles of 30 s at 95 °C, 30 s at 55 °C and 30 s at 72 °C, with a final 10 min extension at 72 °C [12]. Ten microliters of the amplification product was collected and separated on a 1.2 % agarose gel, and detected under UV-light after staining with SYBR^®^ Safe DNA gel stain (Thermo Fisher Scientific). The PCR product was purified with a PCR purification kit (Qiagen) according to the manufacturer’s protocol. The cycle sequencing and sequence reactions were performed by an external company (Dragon Technology Limited, Hong Kong, China). All of the sequences were edited using a Biological Sequence Alignment Editor (Bioedit) and aligned with the ClustalW algorithm found in version 6.0 of the Molecular Evolutionary Genetics Analysis (MEGA 6) software (The Biodesign Institute, Arizona, MA, USA). All of the sequence distances were calculated using the Maximum Parsimony method (Kimura 2-parameter model, bootstrap = 1000 replicates) with MEGA 6.

### Statistical analysis

Peaks above the S/N ratio of the chromatogram were labeled and manually integrated using version 7.2 of the Chromeleon Chromatography Data System software (Thermo Fisher Scientific) to distinguish between the different species of *Acorus*. Principal component analysis (PCA) and Hierarchical clustering analysis (HCA) of the relative peak areas were performed using the SPSS for Windows 16.0 software (SPSS Corporation, Armonk, NY, USA) to differentiate between the different peaks. Summary data were expressed as the mean ± standard deviation (SD) for *n* = 4.

## Results

### Macroscopic features

Sixteen samples of herbs corresponding to ATR, AGR, ACR and AAR were collected from different production sites and numbered from 1 to 16 (Table 1). The different samples were initially identified based on their morphological appearance. The unique features of these samples are described below.

#### ATR

**Fig. 1:**
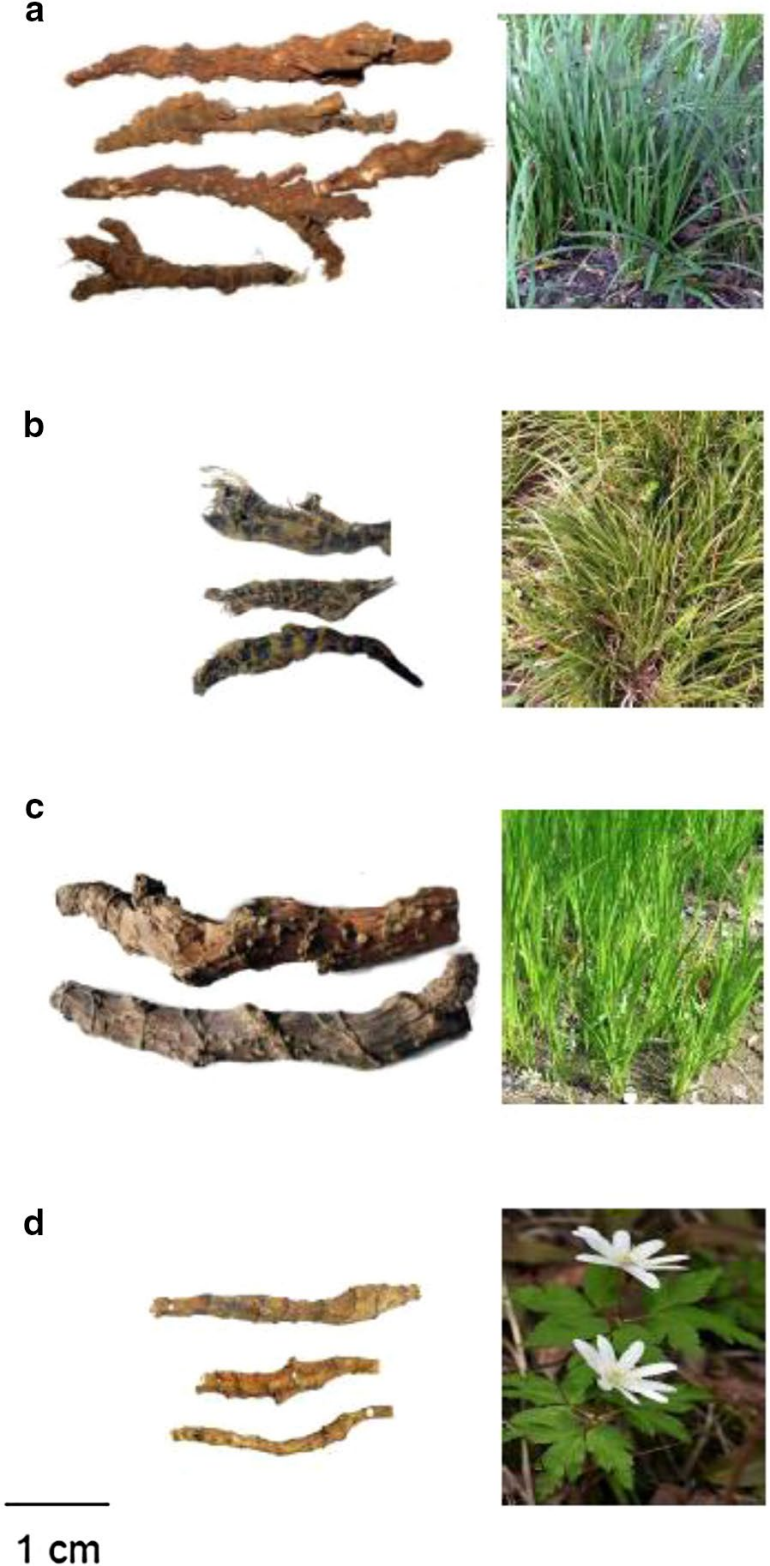
Photographs of the rhizomes of *A. tatarinowii* and its adulterants. **a** *ATR* Acori Tatarinowii Rhizoma; **b** *AGR* Acori Graminei Rhizoma; **c** *ACR* Acori Calami Rhizoma; **d** *AAR* Anemones Altaicae Rhizoma

Compressed-cylindrical, frequently tortuous, normally branched, 3–15 cm in length and 3–10 mm in diameter. Externally brown or greyish-brown, rough, with uneven annulations, internodes of 1–15 mm in length, with fine longitudinal wrinkles, occasionally with remnants of fibrous roots or rounded root scars; leaf scars triangular, arranged alternately, some with hairy and scaly remnants of the leaf bases (Fig. 1a). Odor, aromatic; taste, bitter and slightly pungent.

#### AGR

Almost the same as ATR, but smaller in size. Compressed-cylindrical, frequently tortuous, normally branched, 1–4 cm in length and 2–7 mm in diameter. Externally brown or pale green, rough, with uneven annulations, internodes of 1.5–4 mm long, with fine longitudinal wrinkles, occasionally with remnants of fibrous roots or rounded root scars; leaf scars triangular, arranged alternately, some with hairy and scaly remnants of the leaf bases (Fig. 1b). Odor, aromatic; taste, bitter and slightly pungent.

#### ACR

Similar to ATR, but larger in size. Cylindrical, flattened and branched at the nodes, 5–20 cm in length and 10–15 mm in diameter. Externally, the rhizomes appeared light brown to red, and the inner surface appeared pale brown. Fewer internodes than ATR, 4–15 mm in length with fine longitudinal wrinkles, occasionally with remnants of the fibrous roots or rounded root scars; large triangular leaf scars, arranged alternately, some with hairy and scaly remnants of the leaf bases (Fig. 1c). Strong aromatic odor, pungent taste.

#### AAR

Fusiform in shape, slightly curved, 1–4 cm in length and 3–5 mm in diameter. Externally, yellowish brown to brown in color. Nodes with numerous semi-circular protuberances and root scars (Fig. 1d). Odor slight, slightly sour taste with numbness.

### Microscopic features of transverse sections

#### ATR

**Fig. 2:**
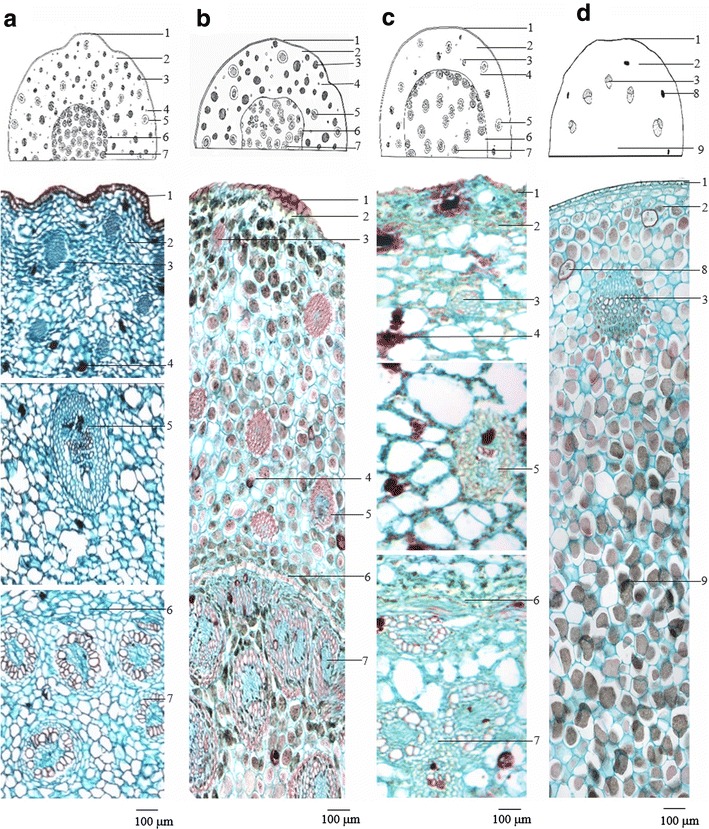
Micrographs show transverse sections of ATR and its adulterants. **a** *ATR* Acori Tatarinowii Rhizoma; **b** *AGR* Acori Graminei Rhizoma; **c** *ACR* Acori Calami Rhizoma; **d** *AAR* Anemones Altaicae Rhizoma. Notations are: *1* epidermis; *2* cortex; *3* fiber bundles; *4* secretory cells; *5* leaf-trace vascular bundle; *6* endodermis; *7* stele vascular bundle; *8* stone cells; *9* pith

Epidermis consisting of one layer of brown cells with a thickened outer wall. Broad cortex with numerous scattered fiber bundles and leaf-trace vascular bundles. Endodermis distinct. Stele vascular bundles amphivasal or collateral, densely lined up in close proximity to the endodermis, gradually becoming larger and sparse going inward, vascular bundles with a distinct sheath. Parenchyma scattered with sub-rounded secretory cells, filled with secretions (Fig. 2a).

#### AGR

Almost the same as ATR, but smaller in size. Epidermis consisting of one layer of brown cells with a thickened outer wall. Broad cortex with numerous scattered fiber bundles and leaf-trace vascular bundles. Endodermis distinct. Stele vascular bundles amphivasal or collateral, densely lined up in close proximity to the endodermis, gradually becoming larger and sparse going inward, vascular bundles sheath distinct. Parenchyma scattered with sub-rounded secretory cells, filled with secretions (Fig. 2b).

#### ACR

Similar to ATR, but larger in size. Epidermis consisting of one layer of brown cells with a thickened outer wall. Broad cortex with numerous scattered fiber bundles and leaf-trace vascular bundles. Fewer fiber bundles compared with ATR. Distinct endodermis. Stele vascular bundles amphivasal or collateral, densely lined up in close proximity to the endodermis, gradually becoming larger and sparse going inward, vascular bundles sheath distinct. Parenchyma scattered with sub-rounded secretory cells, filled with secretions (Fig. 2c).

#### AAR

Epidermis consisting of one layer of flat cells with a thickened outer wall. Sub-spherical stone cells scattered across the outer part of the cortex. Collateral vascular bundles composed of 8–12 units arranged in a ring. Phloem cells flat and shrunken. Cambium indistinct. Xylem vessels polygonal or sub-rounded. Pith large. Parenchymatous cell filled with starch granules (Fig. 2d).

### Microscopic features of powder sections

#### ATR, AGR and ACR

**Fig. 3:**
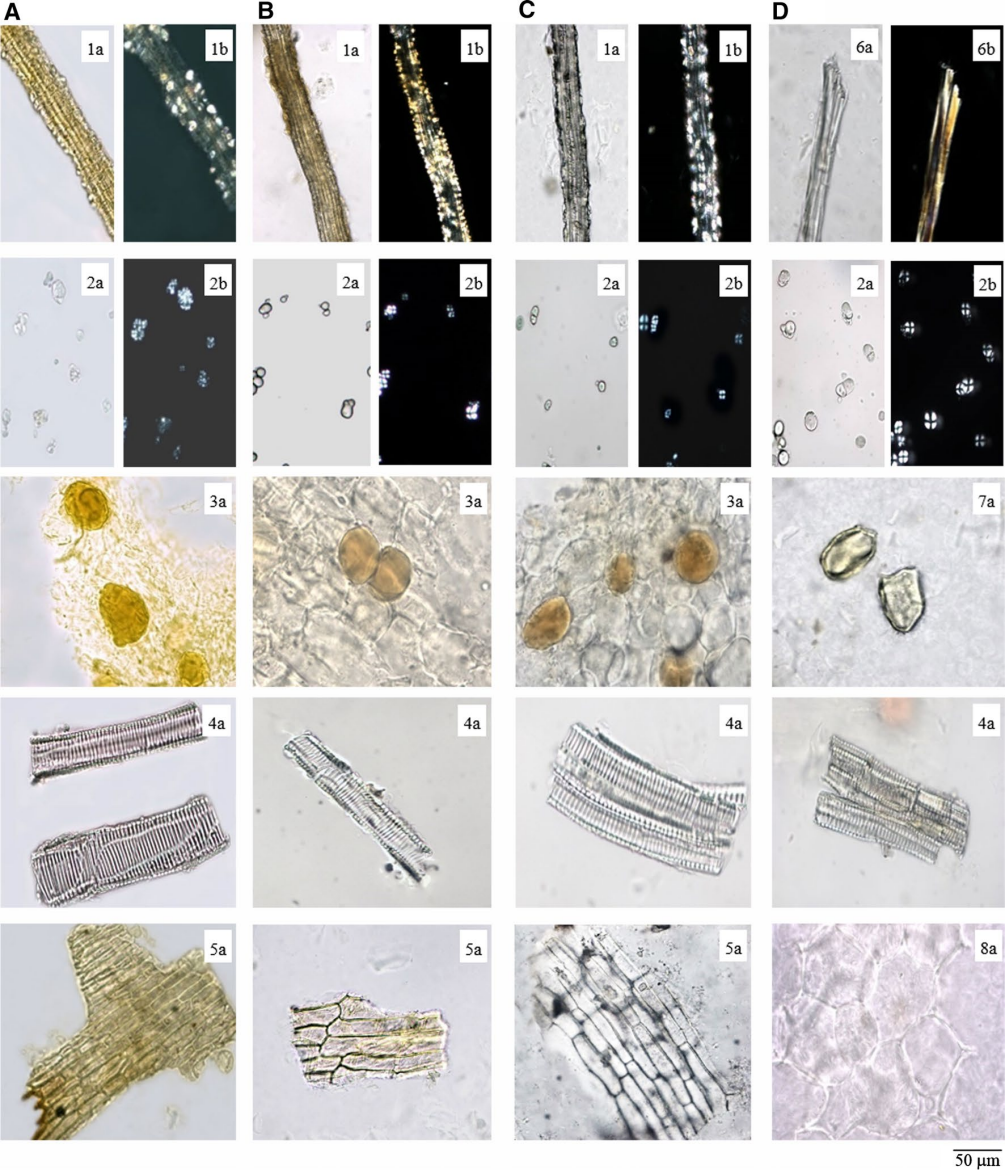
Micrographs show powders of ATR and its adulterants. **A** *ATR* Acori Tatarinowii Rhizoma; **B** *AGR* Acori Graminei Rhizoma; **C** *ACR* Acori Calami Rhizoma; **D** *AAR* Anemones Altaicae Rhizoma. Notations are: *1* crystal fibers; *2* starch granules; *3* secretory cells; *4* vessels; *5* epidermal cells of leaf sheath; *6* fibers; *7* stone cell; *8* parenchymatous cell. *a* Indicated features under the light microscope; *b* Indicate features under the polarized microscope

There were no significant differences in the microscopic features of the ATR, AGR and ACR powders. The fibers were mostly bundled, colorless or pale yellow in color, tapered towards the ends and lignified with distinct pit canals. Fibrous bundles surrounded by cells containing calcium oxalate prisms, which formed crystalline fibers. Polyhedral, sub-polygonal or polycone-like calcium oxalate prisms, which appeared yellowish-white or polychromatic under a polarized microscope. Simple starch granules, which were ellipsoid, spheroidal, long-ovoid, hilum pointed, V-shaped or short slit-shaped with indistinct striation marks; black and cruciate-shaped under a polarized microscope; compound starch granules composed of 2–20 units. Secretory cells abundant, sub-rounded or elongated-rounded, filled with orange-yellow secretions. The epidermal cells of the leaf sheath were greyish-green or pale yellowish-brown and rectangular in shape. The vessels were mainly reticulate (Fig. 3A–C).

#### AAR

Fibers mostly in bundles, yellowish-white or polychromatic under a polarized microscope. Numerous starch granules. Simple starch granules spherical to ovoid, with dotted, cleft or asteroidal hilum, compound granules composed of 2–4 units, black in color and cruciate-shaped under a polarized microscope. Stone cells, which were sub-rounded or oblong in shape. The vessels were mostly reticulate. Parenchymatous cells abundant (Fig. 3D).

Table 2 Characteristics of the ATR, AGR, ACR and AAR samples, as determined by microscopic analysis. These features could serve as parameters for the identification of these herbs.

**Table 2:** Summary of the macroscopic and microscopic features of ATR, AGR, ACR and AAR

	ATR	AGR	ACR	AAR
Morphology				
Shape	Flattish-cylindrical		Cylindrical	Fusiform
Length (cm)	3–15	1–4	5–20	1–4
Diameter (mm)	3–10	2–7	10–15	3–5
Fracture	Fibrous, numerous dotted vascular bundles and brown oil spots visible		Fibrous, numerous dotted vascular bundles visible	Granular protuberances, starchy
Odor	Aromatic; taste bitter and slightly pungent		Strong aromatic and taste pungent	Slight; taste slightly sour and numbness
Transverse section				
1. Epidermis	Sub-square brown cells			Flat square cells
2. Cortex	3. Fiber bundles, 4. Secretory cells and 5. Leaf-trace vascular bundle are scattered			3. Fiber bundles arrange in ring, 8. Stone cells, suborbicular, scattered
	Small intercellular spaces		Large intercellular spaces	
7. Stele vascular bundle	Amphivasal or collateral, densely lined up near the 6. Endodermis			N/A
9. Pith	N/A			Large
Powder				
1. Crystal fibers	Fiber bundles surrounded by cells containing prisms of calcium oxalate, forming crystal fibers			6. Fibers, not crystal fiber
2. Starch granules	Ellipsoid, spheroidal or long-ovoid, black and cruciate-shaped under the polarized microscope			
3. Secretory cells	Abundant, sub-rounded or elongated-rounded			N/A
4. Vessels	Mainly reticulate			
5. Epidermal cells of leaf sheath	Greyish-green or pale yellowish-brown, rectangular			N/A
7. Stone cells	N/A			Abundant
8. Parenchymatous cells	Less			Abundant

The number is referred to the notations in Fig. 2 (transverse section) and Fig. 3 (powder section)

### Chemical analysis by UHPLC-DAD

**Fig. 4:**
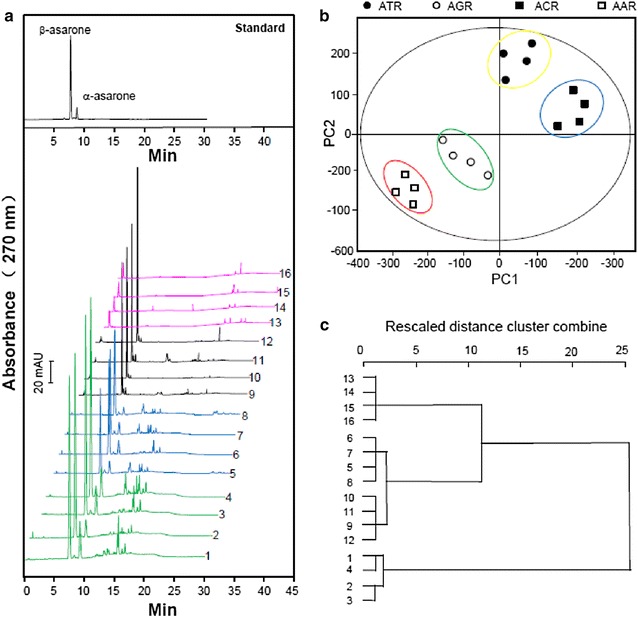
UHPLC-DAD chromatograms of extracts from ATR and its adulterants. **a** UHPLC-DAD chromatograms of 16 samples (number 1–16 for different samples, shown in Table 1) at 270 nm were shown. *Upper panel* shows the *markers*, α-asarone and β-asarone. **b** Score plots for ATR and its adulterants, using peak areas of α-asarone and β-asarone as input data, were shown. **c** Hierarchical clustering analysis for the 16 samples. The loading plot was performed with the original peak areas of α-asarone and β-asarone as input data

α-Asarone and β-asarone, which are the most abundant chemicals in these plant species, were used as chemical markers for ATR. These chemicals have also been reported to exhibit biological functions [13–16]. The samples were analyzed using a DAD detector according to the popular criteria. The detection wavelength of the DAD detector was set to 270 nm to acquire the most abundant peaks. Typical chromatograms of ATR, AGR, ACR and AAR from samples 1–16 are shown in Fig. 4a. The results revealed that there were considerable differences in the chromatographic profiles of the ATR, AGR and ACR extracts compared with the AAR extract. The majority of the α-asarone and β-asarone components were eluted in less than 30 min from the ATR, AGR and ACR extracts. In contrast, the chromatogram of the AAR extract did not contain any α-asarone or β-asarone based on a comparison of its retention times and UV spectra with those of the standard compounds (Fig. 4a). The chromatographic fingerprints of all of the extracts were shown to be highly stable and reproducible using the “Similarity evaluation system for the chromatographic fingerprints of TCM” software, which was developed by the Chinese Pharmacopeia Commission. The application of PCA to the chromatographic fingerprints summarized most of the UHPLC-DAD data into the first two principle components, PC1 and PC2, with two-dimensional score plots showing clear differences between the different samples. It is noteworthy that PC1 and PC2 covered more than 90 % of the total variability. Data for α-asarone and β-asarone were found to be distinct from those of the main cluster profiles of the ATR, AGR, ACR and AAR extracts. The scatter points showed that the samples could be readily classified into four different groups, indicating that it was possible to differentiate between the different plants. The α-asarone and β-asarone contents of the different extracts could therefore be used to discriminate ATR from AGR, ACR and AAR (Fig. 4b). Hierarchical clustering analysis (HCA) was also applied to differentiate between the different extracts using Pearson’s correlation as a measurement (Fig. 4c). In a similar manner to the PCA results, HCA allowed for most of the samples to be classified into two groups, including cluster 1 [samples 1–4 (ATR)] and cluster 2 [samples 5–16 (AGR, ACR and AAR)]. This clustering agreed well with the results of ATR and non-ATR samples. Taken together, these results suggest that ATR can be readily differentiated from the other three *Acorus* species based on differences in their asarone contents. PCA and HCA allowed for the different species to be partially distinguished by visual inspection for conventional quality control purposes.

### DNA analysis by ITS sequence

**Fig. 5:**
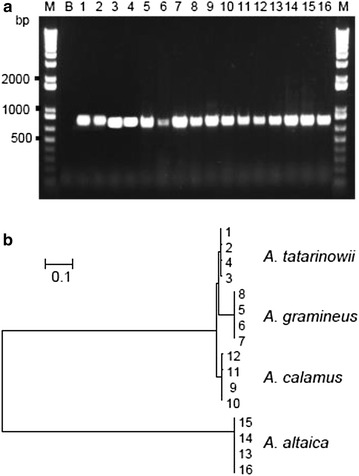
ITS sequence of ATR and its adulterants. **a** Amplicons of ATR and its adulterants with ITS primers. **b** Blank (nuclease-free distilled water); *Lane* 1–4: ATR; *Lane* 5–8: AGR; *Lane* 9–12: ACR; *Lane* 12–16: AAR. All samples produced a band at ~850 bp, *n* = 4. **b** The ITS sequences were identified and matched fully from sequences of GenBank, i.e., *A. tatarinowii* (DQ008851), *A. gramineus* (DQ008849), *A. calamus* (DQ008853) and *A. altaica* (this study). DNA sequences of ITS fragment from 16 samples were submitted to phylogenetic study by Maximum Parsimony method. Two major clades were remarked. The distance corresponding to sequence divergence is indicated by the *bar*

Genomic DNA was isolated from the 16 different samples. The yields for the DNA extraction process were in the range of 50–300 ng of DNA from 50 mg of ATR, AGR, ACR and AAR. Two flanking primers, ITS-S and ITS-AS, were used for the PCR experiments to allow for the identification of the ITS sequences. About 850 base pair were amplified and sequenced in each case (Fig. 5a). The results of the nucleotide sequence analysis experiments (Additional file 1), including the complete ITS1, ITS2 and 5.8S rRNA regions, revealed that the ITS sequences of these samples were highly homologous, regardless of their geographical origins. The ITS sequences of the 16 samples evaluated in the current study were obtained and subjected to phylogenetic analysis using the maximum parsimony method (Fig. 5b). Two major clades were formed, with distance values ranging from 0.572 to 0.605. *A. calamus* formed a sub-clade by itself, whilst another taxon was formed that further divided into two sub-clades representing *A. tatarinowii* and *A. gramineus*. These results therefore demonstrate that the rRNA ITS region can be used to authenticate *Acorus* species.

## Discussion

Substitutes and adulterants of CM could be introduced intentionally or accidentally, not only seriously attenuating therapeutic effects of these materials but also leading to poisoning. The development of an accurate method for the authentication of CM is therefore essential [17]. In this study, ATR and its adulterants were investigated using several promising methods, including macroscopic and microscopic examination, chemical analysis and DNA authentication. The results of the microscopic identification process revealed that AAR could be readily distinguished from the other species by the lack of crystal fibers (specific features of *Acorus* species) in AAR. Moreover, stone cells were only observed in AAR and not in the ATR, AGR or ACR. Morphological identification processes require botanical expertise for the unequivocal authentication of different plant samples because of similarities in the morphological features of related species [18]. Chemical analysis is a commonly used technique for rapid separation, identification and quantification of the chemical components of herbal materials. In this study, the peaks corresponding to α-asarone and β-asarone were used to differentiate between ATR, AGR and ACR, but not AAR. Furthermore, PCA and HCA provided partial visual differentiation according to their species based on these two chemicals.

Macroscopic and microscopic methods are labor intensive, whereas chemical fingerprinting techniques can be adversely affected by external factors. In contrast, genetic tools were more reliable and less demanding in sample amounts and external factors for the authentication of herbal materials at the DNA level. Various DNA-based molecular markers have been developed for the authentication of herbal medicines with an increasing number of applications in recent years [19–24]. ITS was used in the current study as a DNA marker to authenticate the *Acorus* species that were indistinguishable by neither morphological features nor phytochemical compositions. DNA authentication only requires a small amount of sample, exhibits high levels of stability and is largely unaffected by external factors.

This study showed that quality control for the *Acorus* species which was mainly dependent on qualitative characteristics (i.e., morphological authentication, chemical fingerprinting and genetic sequencing) could be used to generate quantitative hypotheses. The combination of quantitative and qualitative methods described in this study provided a new approach to enhancing the research in CM.

## Conclusion

DNA authentication exhibited higher resolution power and reliability than conventional morphological identification and UHPLC in differentiating between different *Acorus* species.

## Additional file

10.1186/s13020-016-0113-x Sequence alignment of the ITS regions and 5.8S rRNA genes from *A. tatarinowii*, *A. gramineus*, *A. calamus* and *A. altaica*. The coding regions are boxed. Primer sequences used for amplification are indicated by arrows. Identical sequences are indicated by (*). Gaps (-) are introduced for the best alignment.

## Abbreviations

ATR: Acori Tatarinowii Rhizoma
AGR: Acori Graminei Rhizoma
ACR: Acori Calami Rhizoma
AAR: Anemones Altaicae Rhizoma
CM: Chinese medicine
DAD: diode-array detector
HCA: hierarchical clustering analysis
ITS: internal transcribed spacer
PCA: principal component analysis
UHPLC: ultra high performance liquid chromatography
