# Supplementary material for: Authentication of Acori Tatarinowii Rhizoma (Shi Chang Pu) and its adulterants by morphological distinction, chemical composition and ITS sequencing
Source: Chin Med. 2016 Sep 26;11:41. doi: 10.1186/s13020-016-0113-x (PMC5037583; doi:10.1186/s13020-016-0113-x)
Supplement: Supplementary file 1 — 10.1186/s13020-016-0113-x Sequence alignment of the ITS regions and 5.8S rRNA genes from A. tatarinowii, A. gramineus, A. calamus and A. altaica. The coding regions are boxed. Primer sequences used for amplification are indicated by arrows. Identical sequences are indicated by (*). Gaps (-) are introduced for the best alignment. [file 13020_2016_113_MOESM1_ESM.pdf]

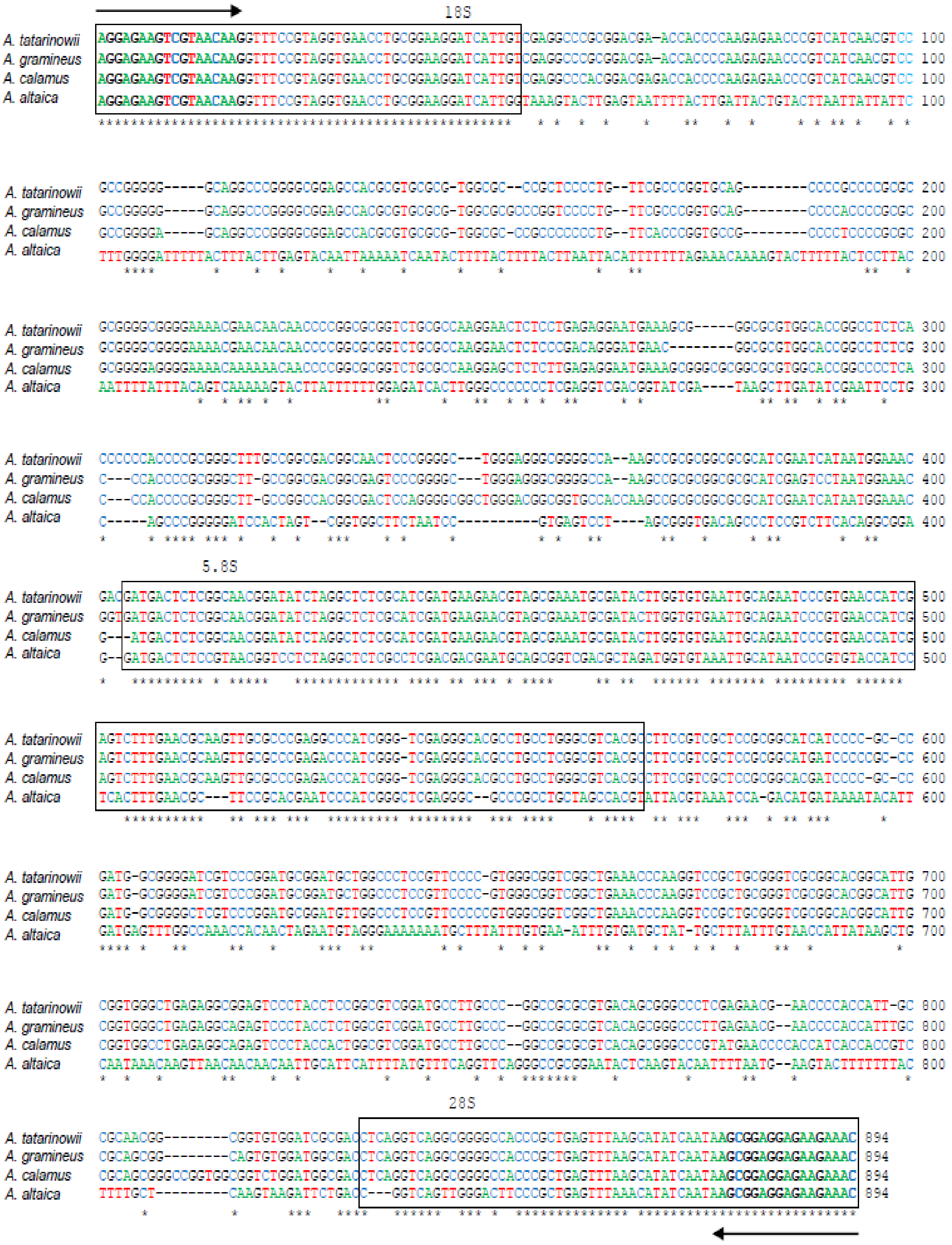
18S
5.8S
28S
A. tatarinowii
A. gramineus
A. calamus
A. altaica
